# Modified hybrid decomposition of the augmented Lagrangian method with larger step size for three-block separable convex programming

**DOI:** 10.1186/s13660-018-1863-z

**Published:** 2018-10-04

**Authors:** Min Sun, Yiju Wang

**Affiliations:** 1 School of Mathematics and Statistics, Zaozhuang University, Zaozhuang, P.R. China; 2 School of Management, Qufu Normal University, Qufu, P.R. China

**Keywords:** The augmented Lagrangian method, Three-block separable convex programming, Step size, Global convergence

## Abstract

The Jacobian decomposition and the Gauss–Seidel decomposition of augmented Lagrangian method (ALM) are two popular methods for separable convex programming. However, their convergence is not guaranteed for three-block separable convex programming. In this paper, we present a modified hybrid decomposition of ALM (MHD-ALM) for three-block separable convex programming, which first updates all variables by a hybrid decomposition of ALM, and then corrects the output by a correction step with constant step size $\alpha \in(0,2-\sqrt{2})$ which is much less restricted than the step sizes in similar methods. Furthermore, we show that $2-\sqrt{2}$ is the optimal upper bound of the constant step size *α*. The rationality of MHD-ALM is testified by theoretical analysis, including global convergence, ergodic convergence rate, nonergodic convergence rate, and refined ergodic convergence rate. MHD-ALM is applied to solve video background extraction problem, and numerical results indicate that it is numerically reliable and requires less computation.

## Introduction

Many problems encountered in applied mathematics area can be formulated as separable convex programming, such as basis pursuit (BP) problem [1–3], video background extraction problem [4–7], image decomposition [8–10], and so on. Thus the solving of separable convex programming plays a fundamental role in applied mathematics and has drawn persistent attention. In the existing literature, several forms of separable convex programming have been investigated [11–15], in which the following three-block separable convex programming rouses more interest:

1 $$ \min \Biggl\{ \sum_{i=1}^{3} \theta_{i}(x_{i})\Big|\sum_{i=1}^{3}A_{i}x_{i}=b,x_{i} \in\mathcal{X}_{i},i=1,2,3 \Biggr\} , $$

where $\theta_{i}: \mathcal{R}^{n_{i}}\mapsto(-\infty,+\infty]$ ($i=1,2,3$) are lower semicontinuous proper convex functions, $A_{i}\in\mathcal {R}^{l\times n_{i}}$ ($i=1,2,3$) and $b\in\mathcal{R}^{l}$, $\mathcal{X}_{i}$ ($i=1,2,3$) are nonempty closed convex sets in $\mathcal{R}^{n_{i}}$ ($i=1,2,3$). Throughout this paper, we assume that the solution set of problem (1) is nonempty.

The Lagrangian and augmented Lagrangian functions of problem (1) are defined, respectively, as

2 $$\begin{aligned}& \mathcal{L}(x_{1},x_{2},x_{3}, \lambda)=\sum_{i=1}^{3}\theta_{i}(x_{i})- \Biggl\langle \lambda, \sum_{i=1}^{3}A_{i}x_{i}-b \Biggr\rangle , \end{aligned}$$

3 $$\begin{aligned}& \mathcal{L}_{\beta}(x_{1},x_{2},x_{3}, \lambda )=\mathcal{L}(x_{1},x_{2},x_{3},\lambda)+ \frac{\beta}{2} \Biggl\Vert \sum_{i=1}^{3}A_{i}x_{i}-b \Biggr\Vert ^{2}, \end{aligned}$$

where $\lambda\in\mathcal{R}^{l}$ is the Lagrange multiplier associated with the linear constraints in (1), and $\beta>0$ is a penalty parameter. Applying the augmented Lagrangian method (ALM) [16] to problem (1), we can obtain the following iterative scheme:

4 $$ \textstyle\begin{cases} (x_{1}^{k+1},x_{2}^{k+1},x_{3}^{k+1})=\operatorname{argmin}\{\mathcal{L}_{\beta}(x_{1},x_{2},x_{3},\lambda^{k})|x_{1}\in\mathcal{X}_{1},x_{2}\in\mathcal{X}_{2},x_{3}\in \mathcal{X}_{3}\}, \\ \lambda^{k+1}=\lambda^{k}-\beta(A_{1}x_{1}^{k+1}+A_{2}x_{2}^{k+1}+A_{3}x_{3}^{k+1}-b). \end{cases} $$

Obviously, three variables $x_{1}$, $x_{2}$, $x_{3}$ are all involved in the minimization problem of (4), which makes the method often hard to implement. One technique to handle this is to split the subproblem into several small scale subproblems. Based on this, if we split it in a Gauss–Seidel manner and adopt the famous alternating direction method of multiplier (ADMM) [11], we obtain the following iterative scheme:

5 $$ \textstyle\begin{cases} x_{1}^{k+1}=\operatorname{argmin}\{\mathcal{L}_{\beta}(x_{1},x_{2}^{k},x_{3}^{k},\lambda ^{k})|x_{1}\in\mathcal{X}_{1}\}, \\ x_{2}^{k+1}=\operatorname{argmin}\{\mathcal{L}_{\beta}(x_{1}^{k+1},x_{2},x_{3}^{k},\lambda ^{k})|x_{2}\in\mathcal{X}_{2}\}, \\ x_{3}^{k+1}=\operatorname{argmin}\{\mathcal{L}_{\beta}(x_{1}^{k+1},x_{2}^{k+1},x_{3},\lambda^{k})|x_{3}\in\mathcal{X}_{3}\}, \\ \lambda^{k+1}=\lambda^{k}-\beta(A_{1}x_{1}^{k+1}+A_{2}x_{2}^{k+1}+A_{3}x_{3}^{k+1}-b). \end{cases} $$

On the other hand, if we split it in a Jacobian manner, we get the following full parallel iterative scheme:

6 $$ \textstyle\begin{cases} x_{1}^{k+1}=\operatorname{argmin}\{\mathcal{L}_{\beta}(x_{1},x_{2}^{k},x_{3}^{k},\lambda ^{k})|x_{1}\in\mathcal{X}_{1}\}, \\ x_{2}^{k+1}=\operatorname{argmin}\{\mathcal{L}_{\beta}(x_{1}^{k},x_{2},x_{3}^{k},\lambda ^{k})|x_{2}\in\mathcal{X}_{2}\}, \\ x_{3}^{k+1}=\operatorname{argmin}\{\mathcal{L}_{\beta}(x_{1}^{k},x_{2}^{k},x_{3},\lambda ^{k})|x_{3}\in\mathcal{X}_{3}\}, \\ \lambda^{k+1}=\lambda^{k}-\beta(A_{1}x_{1}^{k+1}+A_{2}x_{2}^{k+1}+A_{3}x_{3}^{k+1}-b). \end{cases} $$

Compared with the minimization problem in (4), the scale of the minimization procedures in (5) and (6) is decreased, and they fully utilize the separable property of the objective function of (1), thus the new iterative schemes (5) and (6) gain some solvability. However, their convergence cannot be guaranteed under milder conditions as shown in [12, 17]. To overcome this drawback, several new techniques, such as the regularization method with large proximal parameter [18–23], the prediction-correction method with shrunk step size [12, 13, 24–26], etc., have been developed.

Compared with the regularization method, the prediction-correction method has attracted extensive interest, and during the past decades many scholars have performed studies in this direction. For example, He *et al.* [24] proposed an ADMM-based contraction type method for solving multi-block separable convex programming, which first generates a temporal iterate by (5), and then corrects it with a Gaussian back substitution procedure. Later, He *et al.* [12] developed a full Jacobian decomposition of the augmented Lagrangian method for solving multi-block separable convex programming, which first generates a temporal iterate by (6), and then corrects it with a constant step size or varying step size. Different from the above, Han *et al.* [13] proposed a partial splitting augmented Lagrangian method for solving three-block separable convex programming, which first updates the primal variables $x_{1}$, $x_{2}$, $x_{3}$ in a partially-parallel manner, and then corrects $x_{3}$, *λ* with a constant step size. Later, Wang *et al.* [25] presented a proximal partially-parallel splitting method for solving multi-block separable convex programming, which first updates all primal variables in a partially-parallel manner, and then corrects the output with a constant step size or varying step size. Quite recently, Chang *et al.* [26] proposed a convergent prediction-correction-based ADMM in which more minimization problems are involved. In conclusion, the above iteration schemes first generate a temporal iterate by (5) or (6) or their variants, and then generate the new iterate by correcting the temporal iterate with varying step size or a constant step size.

Varying step size needs to be dynamically updated at each iteration, which might be computationally demanding for large-scale (1). Hence in this paper, we consider the prediction-correction method with constant step size for solving problem (1). To the best of our knowledge, He *et al.* [12] first proposed a prediction-correction method with constant step size for solving (1), and they proved that the upper bound of the constant step size is 0.2679. By taking a hybrid splitting of (4) as the prediction step, Wang *et al.* [25] relaxed the upper bound of the constant step size to 0.3670 and Han *et al.* [13] further relaxed it to 0.3820. In practice, to enhance the numerical efficiency of the corresponding iteration method, larger values of the step size are preferred as long as the convergence is still guaranteed [26]. In this paper, based on the methods in [12, 13, 25], we propose a modified hybrid decomposition of the augmented Lagrangian method with constant step size, whose upper bound is relaxed to 0.5858.

The rest of this paper is organized as follows. Section 2 lists some notations and basic results. In Sect. 3, we present a modified hybrid decomposition of the augmented Lagrangian method with larger step size for problem (1) and establish its global convergence and refined convergence rate. Furthermore, a simple example is given to illustrate that $2-\sqrt{2}\cong0.5858$ is the optimal upper bound of the constant step size in MHD-ALM. In Sect. 4, some numerical results are given to demonstrate the numerical advantage of larger step size. Finally, a brief conclusion including some possible future works is drawn in Sect. 5.

## Preliminaries

In this section, we give some notations and basic results about the minimization problem (1), which will be used in the forthcoming discussions.

Throughout this paper, we define the following notations:

$$\begin{aligned}& x=(x_{1},x_{2},x_{3}),\qquad v=(x_{2},x_{3}, \lambda),\qquad w=(x_{1},x_{2},x_{3},\lambda), \\& \theta(x)=\theta_{1}(x_{1})+\theta_{2}(x_{2})+ \theta_{3}(x_{3}) \end{aligned}$$

and

$$\mathcal{A}=(A_{1},A_{2},A_{3}),\qquad \mathcal{X}=\mathcal{X}_{1}\times\mathcal {X}_{2}\times \mathcal{X}_{3},\qquad \mathcal{V}=\mathcal{X}_{2}\times \mathcal {X}_{3}\times\mathcal{R}^{l},\qquad \mathcal{W}= \mathcal{X}\times\mathcal{R}^{l}. $$

### Definition 2.1

A tuple $(x^{*},\lambda^{*})\in\mathcal{W}$ is called a saddle point of the Lagrangian function (2) if it satisfies the inequalities

7 $$ \mathcal{L}_{\lambda\in\mathcal {R}^{l}}\bigl(x^{*},\lambda\bigr)\leq\mathcal{L} \bigl(x^{*},\lambda^{*}\bigr)\leq\mathcal{L}_{x\in \mathcal{X}}\bigl(x,\lambda^{*}\bigr). $$

Solving problem (1) is equivalent to finding a saddle point of $\mathcal{L}(x,\lambda)$ [26, 27]. Therefore, to solve (1), we only need to solve the two inequalities in (7), which can be written as the following mixed variational inequality:

8 $$ \theta(x)-\theta\bigl(x^{*}\bigr)+\bigl(w-w^{*}\bigr)^{\top}F \bigl(w^{*}\bigr)\geq0, \quad \forall w\in\mathcal{W}, $$

where

9 $$ F(w)=\left ( \textstyle\begin{array}{@{}c@{}} -A_{1}^{\top}\lambda\\-A_{2}^{\top}\lambda\\-A_{3}^{\top}\lambda\\ {\sum_{i=1}^{3}A_{i}x_{i}}-b \end{array}\displaystyle \right ) =\left ( \textstyle\begin{array}{c} -\mathcal{A}^{\top}\lambda\\\mathcal{A}x-b \end{array}\displaystyle \right ) =\left ( \textstyle\begin{array}{@{}c@{\quad}c@{}} 0&-\mathcal{A}^{\top}\\\mathcal{A}&0 \end{array}\displaystyle \right )\left ( \textstyle\begin{array}{@{}c@{}} x\\\lambda \end{array}\displaystyle \right )-\left ( \textstyle\begin{array}{@{}c@{}} 0\\b \end{array}\displaystyle \right ). $$

Because $F(w)$ is a linear mapping with skew-symmetric coefficient matrix, it satisfies the following property:

10 $$ \bigl(w'-w\bigr)^{\top}F \bigl(w'\bigr)=\bigl(w'-w\bigr)^{\top}F(w), \quad \forall w',w\in\mathcal{W}. $$

The mixed variational inequality (8) is denoted by $\operatorname{MVI}(\mathcal{W},F,\theta)$, whose solution set is denoted by $\mathcal {W}^{*}$, which is nonempty from the assumption on problem (1).

To solve $\operatorname{MVI}(\mathcal{W},F,\theta)$, He *et al.* [28] presented the following prototype algorithm:

**A prototype algorithm for** $\boldsymbol{\operatorname{MVI}(\mathcal{W},F,\theta)}$ **, denoted by ProAlo:**

*Prediction*: For given $v^{k}$, find $\hat{w}^{k}\in\mathcal{W}$ and *Q* satisfying

11 $$ \theta(x)-\theta\bigl(\hat{x}^{k}\bigr)+\bigl(w-\hat {w}^{k}\bigr)^{\top}F\bigl(\hat{w}^{k}\bigr)\geq \bigl(v-\hat{v}^{k}\bigr)^{\top}Q\bigl(v^{k}- \hat{v}^{k}\bigr),\quad \forall w\in\mathcal{W}, $$

where the matrix *Q* has the property: $(Q+Q^{\top})$ is positive definite.

*Correction*: Determine a nonsingular matrix *M*, a scalar $\alpha >0$, and generate the new iterate $v^{k+1}$ via

12 $$ {v}^{k+1}=v^{k}-\alpha M\bigl(v^{k}- \hat{v}^{k}\bigr). $$

### Condition 2.1

The matrices *Q*, *M* in the ProAlo satisfy that the three matrices $Q+Q^{\top}$, $H:=QM^{-1}$, $G(\alpha):=Q+Q^{\top}-\alpha M^{\top}HM$ are positive definite.

Under Condition 2.1, He *et al.* [28] established the convergence results of ProAlo, including the global convergence, the worst-case $\mathcal {O}(1/t)$ convergence rate in ergodic or nonergodic sense, where *t* is the iteration counter. See Theorems 3.3, 4.2, 4.5 in [28].

To end this section, we give the following lemma which will be used in the subsequent section.

### Lemma 2.1

([27])

*Let* $\mathcal{X}\subseteq\mathcal{R}^{n}$ *be a closed nonempty convex set*, $\theta(x)$ *and* $f(x)$ *be two convex functions*. *If the function* $\theta(x)$ *is nondifferentiable*, *the function* $f(x)$ *is differentiable*, *and the solution set of the problem* $\min\{\theta(x)+f(x)|x\in\mathcal{X}\}$ *is nonempty*, *then*

$$x^{*}\in\operatorname{argmin}\bigl\{ \theta(x)+f(x)|x\in\mathcal{X}\bigr\} $$

*if and only if*

$$x^{*}\in\mathcal{X},\quad \theta(x)-\theta\bigl(x^{*}\bigr)+\bigl(x-x^{*} \bigr)^{\top}\nabla f\bigl(x^{*}\bigr)\geq 0,\quad \forall x\in\mathcal{X}. $$

## Algorithm and its convergence

In this section, we give the process of the modified hybrid decomposition of the augmented Lagrangian method (MHD-ALM) for three-block separable convex programming (1) and establish its convergence results, including global convergence, ergodic convergence rate, nonergodic convergence rate, and refined ergodic convergence rate.

**Algorithm: MHD-ALM**

*Step* 0.: Let parameters $\alpha\in(0,2-\sqrt{2})$, $\beta>0$, tolerance error $\varepsilon>0$. Choose an initial point $v^{0}=(x_{2}^{0},x_{3}^{0},\lambda^{0})\in\mathcal{V}$. Set $k=0$.
*Step* 1.: Compute the prediction iterate $\tilde{w}^{k}=(\tilde {x}_{1}^{k},\tilde{x}_{2}^{k},\tilde{x}_{3}^{k},\tilde{\lambda}^{k})$ via
  13 $$ \textstyle\begin{cases} \tilde{x}_{1}^{k}= {\operatorname{argmin}}\{\mathcal{L}_{\beta}(x_{1},x_{2}^{k},x_{3}^{k},\lambda^{k})|x_{1}\in\mathcal{X}_{1}\}, \\ \tilde{x}_{2}^{k}= {\operatorname{argmin}}\{\mathcal{L}_{\beta}(\tilde {x}_{1}^{k},x_{2},x_{3}^{k},\lambda^{k})|x_{2}\in\mathcal{X}_{2}\}, \\ \tilde{x}_{3}^{k}= {\operatorname{argmin}}\{\mathcal{L}_{\beta}(\tilde {x}_{1}^{k},x_{2}^{k},x_{3},\lambda^{k})|x_{3}\in\mathcal{X}_{3}\}, \\ \tilde{\lambda}^{k}=\lambda^{k}-\beta(A_{1}\tilde{x}_{1}^{k}+A_{2}\tilde {x}_{2}^{k}+A_{3}\tilde{x}_{3}^{k}-b). \end{cases} $$
*Step* 2.: If $\max\{\|A_{2}x_{2}^{k}-A_{2}\tilde{x}_{2}^{k}\|,\| A_{3}x_{3}^{k}-A_{3}\tilde{x}_{3}^{k}\|,\|\lambda^{k}-\tilde{\lambda}^{k}\|\}\leq \varepsilon$, then stop; otherwise, go to Step 3.
*Step* 3.: Generate the new iterate ${w}^{k+1}=({x}_{1}^{k+1},{x}_{2}^{k+1},{x}_{3}^{k+1},{\lambda}^{k+1})$ by
  14 $$ \textstyle\begin{cases} {x}_{1}^{k+1}=\tilde{x}_{1}^{k}, \\ {x}_{2}^{k+1}=x_{2}^{k}-\alpha(x_{2}^{k}-\tilde{x}_{2}^{k}), \\ {x}_{3}^{k+1}=x_{3}^{k}-\alpha(x_{3}^{k}-\tilde{x}_{3}^{k}), \\ {\lambda}^{k+1}=\lambda^{k}-\alpha(\lambda^{k}-\tilde{\lambda}^{k}). \end{cases} $$
  Replace $k+1$ by *k*, and go to Step 1.

### Remark 3.1

Different from the iterative schemes (5) and (6), the iterative scheme (13) first updates the primal variable $x_{1}$ and then updates the primal variables $x_{2}$, $x_{3}$ in a parallel manner. Furthermore, the feasible set of the step size *α* in MHD-ALM is extended from $(0,0.2679)$ in [12], $(0,0.3820)$ in [13], $(0,0.3670)$ in [25] to $(0,0.5858)$.

The methods in [12, 13, 24–26] and MHD-ALM all fall into the algorithmic framework of prediction-correction methods. The main differences among these methods are: (i) in the prediction step, the methods in [24, 26] update all the primal variables in a sequential order; the method in [12] updates all the primal variables in a parallel manner; the methods in [13, 25] and MHD-ALM update all the primal variables in a partial parallel manner, i.e., they first update $x_{1}$ and then update $x_{2}$, $x_{3}$ in a parallel manner; (ii) in the correction step, the method in [13] updates $x_{3}$, *λ*; the method in [26] and MHD-ALM update $x_{2}$, $x_{3}$, *λ*, and the methods in [12, 24, 25] update all the variables.

The convergence analysis of MHD-ALM needs the following assumption and auxiliary sequence.

### Assumption 3.1

The matrices $A_{2}$, $A_{3}$ in problem (1) are both full column rank.

Define an auxiliary sequence $\hat{w}^{k}=(\hat{x}_{1}^{k},\hat{x}_{2}^{k},\hat {x}_{3}^{k},\hat{\lambda}^{k})$ as

15 $$ \hat{x}_{i}^{k}=\tilde{x}_{i}^{k} \quad (i=1,2,3),\qquad \hat{\lambda}^{k}=\lambda^{k}-\beta \bigl(A_{1}\tilde{x}_{1}^{k}+A_{2}{x}_{2}^{k}+A_{3}{x}_{3}^{k}-b \bigr). $$

To prove the convergence results of MHD-ALM, we only need cast it into the ProAlo and ensure the following two conditions hold: (i) the generated sequence satisfying (11), (12); (ii) the resulting matrices *Q*, *M* satisfying Condition 2.1 in Sect. 2. We first verify the first condition. Based on Lemma 2.1, we can derive the first order optimality conditions of the subproblems in (13), which are summarized in the following lemma.

### Lemma 3.1

*Let* $\{w^{k}\}$ *be the sequence generated by MHD*-*ALM and* $\{\hat{w}^{k}\}$ *be defined as in* (15). *Then it holds that*

16 $$ \theta(x)-\theta\bigl(\hat{x}^{k}\bigr)+\bigl(w- \hat{w}^{k}\bigr)^{\top}F\bigl(\hat{w}^{k}\bigr)\geq \bigl(v-\hat {v}^{k}\bigr)^{\top}Q\bigl(v^{k}- \hat{v}^{k}\bigr), \quad \forall w\in\mathcal{W}, $$

*where the matrix* *Q* *is defined by*

17 $$Q=\left(\begin{array}{ccc} \beta A_{2}^{⊤}A_{2} & 0 & 0\\ 0 & \beta A_{3}^{⊤}A_{3} & 0\\ -A_{2} & -A_{3} & I_{l}/\beta \end{array}\right).$$

### Proof

Based on Lemma 2.1 and using the notation of $\hat{w}^{k}$ in (15), the first order optimality conditions for the three minimization problems in (13) can be summarized as the following inequalities:

$$\begin{aligned}& \theta_{1}(x_{1})-\theta_{1}\bigl( \hat{x}_{1}^{k}\bigr)+\bigl(x_{1}- \hat{x}_{1}^{k}\bigr)^{\top}\bigl(-A_{1}^{\top}\hat{\lambda}^{k}\bigr)\geq0,\quad \forall x_{1}\in \mathcal{X}_{1}, \\& \theta_{2}( {x_{2}})-\theta_{2}\bigl( \hat{x}_{2}^{k}\bigr)+\bigl(x_{2}- \hat{x}_{2}^{k}\bigr)^{\top}\bigl(-A_{2}^{\top}\hat{\lambda}^{k}-\beta A_{2}^{\top}A_{2} \bigl(x_{2}^{k}-\hat{x}_{2}^{k}\bigr) \bigr)\geq0,\quad \forall x_{2}\in\mathcal{X}_{2}, \\& \theta_{3}(x_{3})-\theta_{3}\bigl( \hat{x}_{3}^{k}\bigr)+\bigl(x_{3}- \hat{x}_{3}^{k}\bigr)^{\top}\bigl(-A_{3}^{\top}\hat{\lambda}^{k}-\beta A_{3}^{\top}A_{3} \bigl(x_{3}^{k}-\hat{x}_{3}^{k}\bigr) \bigr)\geq0,\quad \forall x_{3}\in\mathcal{X}_{3}. \end{aligned}$$

Furthermore, the definition of the variable $\hat{\lambda}^{k}$ in (15) gives

$$\begin{aligned}& \bigl(\lambda-\hat{\lambda}^{k}\bigr)^{\top}\biggl(A_{1}\hat{x}_{1}^{k}+A_{2} \hat{x}_{2}^{k}+A_{3}\hat {x}_{3}^{k}-b+A_{2} \bigl(x_{2}^{k}-\hat{x}_{2}^{k} \bigr)+A_{3}\bigl(x_{3}^{k}-\hat{x}_{3}^{k} \bigr)+\frac{1}{\beta }\bigl(\hat{\lambda}^{k}-\lambda^{k} \bigr) \biggr)=0, \\& \quad \forall\lambda\in\mathcal{R}^{l}. \end{aligned}$$

Adding the above four inequalities, rearranging terms, and using the definition of the matrix *Q*, the function $F(w)$, we can get the result (16). This completes the proof. □

### Remark 3.2

When $\max\{\|A_{2}x_{2}^{k}-A_{2}\tilde{x}_{2}^{k}\|, \| A_{3}x_{3}^{k}-A_{3}\tilde{x}_{3}^{k}\|, \|\lambda^{k}-\tilde{\lambda}^{k}\|\}=0$, by (15), we get $A_{2}x_{2}^{k}=A_{2}\hat{x}_{2}^{k}$, $A_{3}x_{3}^{k}=A_{3}\hat {x}_{3}^{k}$, $\lambda^{k}=\hat{\lambda}^{k}$. Thus, $Q(v^{k}-\hat{v}^{k})=0$. This and inequality (16) indicate that

$$\theta(x)-\theta\bigl(\hat{x}^{k}\bigr)+\bigl(w-\hat{w}^{k} \bigr)^{\top}F\bigl(\hat{w}^{k}\bigr)\geq0,\quad \forall w\in \mathcal{W}. $$

Therefore, $w^{k}\in\mathcal{W}^{*}$, and the stopping criterion of MHD-ALM is reasonable.

By the definition of $\hat{\lambda}^{k}$ in (15), the updating formula of $\tilde{\lambda}^{ {k}}$ can be represented as

$$\begin{aligned} \tilde{\lambda}^{k} =&\lambda^{k}-\beta \bigl(A_{1}\tilde{x}_{1}^{k}+A_{2} \tilde {x}_{2}^{k}+A_{3}\tilde{x}_{3}^{k}-b \bigr) \\ =&\lambda^{k}- \bigl(\lambda^{k}-\hat{\lambda}^{k}- \beta\bigl(A_{2}\bigl(x_{2}^{k}-\hat {x}_{2}^{k}\bigr)+A_{3}\bigl(x_{3}^{k}- \hat{x}_{3}^{k}\bigr)\bigr) \bigr) \\ =&\lambda^{k}-(-\beta A_{2},-\beta A_{3},I_{l}) \bigl(v^{k}-\hat{v}^{k}\bigr). \end{aligned}$$

This together with (14), (15) gives

18 $$ \textstyle\begin{cases} {x}_{1}^{k+1}=\tilde{x}_{1}^{k}, \\ {v}^{k+1}=v^{k}-\alpha M(v^{k}-\hat{v}^{k}), \end{cases} $$

where the matrix *M* is defined as

19 $$M=\left(\begin{array}{ccc} I_{l} & 0 & 0\\ 0 & I_{l} & 0\\ -\beta A_{2} & -\beta A_{3} & I_{l} \end{array}\right).$$

Now to establish the convergence results of MHD-ALM, we only need to verify that the matrices *Q*, *M* satisfy Condition 2.1 in Sect. 2.

### Lemma 3.2

*Let the matrices* *Q*, *M* *be defined as in* (17) *and* (19). *If* $\alpha\in(0,0.5858)$ *and Assumption *3.1 *hold*, *then we have*

(i) *the symmetric matrix* $Q+Q^{\top}$ *is positive definite*;
(ii) *the matrix* $H=QM^{-1}$ *is symmetric and positive definite*;
(iii) *the matrix* $G(\alpha)=Q+Q^{\top}-\alpha M^{\top}HM$ *is symmetric and positive definite*.

### Proof

(i) From the definition of *Q*, we have

$$Q+Q^{⊤}=\left(\begin{array}{ccc} 2\beta A_{2}^{⊤}A_{2} & 0 & -A_{2}^{⊤}\\ 0 & 2\beta A_{3}^{⊤}A_{3} & -A_{3}^{⊤}\\ -A_{2} & -A_{3} & 2I_{l}/\beta \end{array}\right).$$

Therefore, for any $v=(x_{2},x_{3},\lambda)\neq0$, we have

20 $$ v^{\top}\bigl(Q+Q^{\top}\bigr)v =2\beta \|A_{2}x_{2}\|^{2}+2\beta\|A_{3}x_{3} \|^{2}+2\lambda^{\top}(A_{2}x_{2}+A_{3}x_{3})+ \frac{2}{\beta}\|\lambda\|^{2}. $$

- If $(x_{2},x_{3})=0$, then $\lambda\neq0$, so by (20), we get $v^{\top}(Q+Q^{\top})v=\frac{2}{\beta}\|\lambda\|^{2}>0$.
- If $(x_{2},x_{3})\neq0$, then from (20) we get
  $$v^{\top}\bigl(Q+Q^{\top}\bigr)v\geq\beta\|A_{2}x_{2} \|^{2}+\beta\|A_{3}x_{3}\|^{2}>0, $$
  where the first inequality follows from the inequality $2x^{\top}y\geq -(\beta\|x\|^{2}+\|y\|^{2}/\beta)$, and the second inequality comes from Assumption 3.1.

(ii) From the definition of *Q*, *M*, we have

$$H=\left(\begin{array}{ccc} \beta A_{2}^{⊤}A_{2} & 0 & 0\\ 0 & \beta A_{3}^{⊤}A_{3} & 0\\ 0 & 0 & I_{l}/\beta \end{array}\right),$$

which is obviously positive definite by Assumption 3.1.

(iii) Similarly, from the definition of *Q*, *M*, we have

$$\begin{array}{rcl} G(\alpha ) & = & \left(\begin{array}{ccc} \beta (2-2\alpha )A_{2}^{⊤}A_{2} & -\alpha \beta A_{2}^{⊤}A_{3} & -(1-\alpha )A_{2}^{⊤}\\ -\alpha \beta A_{3}^{⊤}A_{2} & \beta (2-2\alpha )A_{3}^{⊤}A_{3} & -(1-\alpha )A_{3}^{⊤}\\ -(1-\alpha )A_{2} & -(1-\alpha )A_{3} & (2-\alpha )I_{l}/\beta \end{array}\right)\\ & = & L^{⊤}R(\alpha )L, \end{array}$$

where

$$L=\left(\begin{array}{ccc} \sqrt{\beta }A_{2} & 0 & 0\\ 0 & \sqrt{\beta }A_{3} & 0\\ 0 & 0 & I_{l}/\sqrt{\beta } \end{array}\right),\;R(\alpha )=\left(\begin{array}{ccc} (2-2\alpha )I_{l} & -\alpha I_{l} & -(1-\alpha )I_{l}\\ -\alpha I_{l} & (2-2\alpha )I_{l} & -(1-\alpha )I_{l}\\ -(1-\alpha )I_{l} & -(1-\alpha )I_{l} & (2-\alpha )I_{l} \end{array}\right).$$

This together with Assumption 3.1 implies that we only need to prove the matrix ${R(\alpha)}$ is positive definite. In fact, it can be written as

$$R(\alpha )=\left(\begin{array}{ccc} 2-2\alpha & -\alpha & -(1-\alpha )\\ -\alpha & 2-2\alpha & -(1-\alpha )\\ -(1-\alpha ) & -(1-\alpha ) & 2-\alpha \end{array}\right)⊗I_{l},$$

where ⊗ denotes the matrix Kronecker product. Thus, we only need to prove the 3 order matrix

$$\left(\begin{array}{ccc} 2-2\alpha & -\alpha & -(1-\alpha )\\ -\alpha & 2-2\alpha & -(1-\alpha )\\ -(1-\alpha ) & -(1-\alpha ) & 2-\alpha \end{array}\right)$$

is positive definite, whose three eigenvalues are $\lambda_{1}=2-\alpha $, $\lambda_{2}=2 - 2\alpha- \sqrt{3 \alpha^{2} - 4 \alpha+ 2}$, $\lambda_{3}=2-2\alpha+\sqrt{3 \alpha^{2} - 4 \alpha+ 2}$. Then, solving the following three inequalities simultaneously

$$\textstyle\begin{cases} 2-\alpha>0, \\ 2 - 2\alpha- \sqrt{3 \alpha^{2} - 4 \alpha+ 2}>0, \\ 2-2\alpha+\sqrt{3 \alpha^{2} - 4 \alpha+ 2}>0, \end{cases} $$

we get $0<\alpha<2-\sqrt{2}\cong0.5858$. Therefore, the matrix *G* is positive definite for any $\alpha\in(0,0.5858)$. This completes the proof. □

Lemma 3.2 indicates that the matrices *Q*, *M* defined as in (17) and (19) satisfy Condition 2.1 in Sect. 2, and thus we get the following convergence results of MHD-ALM based on Theorems 3.3, 4.2, 4.5 in [28].

### Theorem 3.1

(Global convergence)

*Let* $\{w^{k}\}$ *be the sequence generated by MHD*-*ALM*. *Then it converges to a vector* $w^{\infty}$, *which belongs to* $\mathcal{W}^{*}$.

### Theorem 3.2

(Ergodic convergence rate)

*Let* $\{w^{k}\}$ *be the sequence generated by MHD*-*ALM*, $\{\hat{w}^{k}\}$ *be the corresponding sequence defined in* (15). *Set*

$$\bar{w}^{t}=\frac{1}{t}\sum_{k=0}^{t} \hat{w}^{k}. $$

*Then*, *for any integer* $t\geq1$, *we have*

21 $$ \theta\bigl(\bar{x}^{t}\bigr)-\theta(x)+\bigl(\bar {w}^{t}-w\bigr)^{\top}F(w)\leq\frac{1}{2\alpha t} \bigl\Vert v-v^{0} \bigr\Vert _{H}^{2},\quad \forall w\in \mathcal{W}. $$

### Theorem 3.3

(Nonergodic convergence rate)

*Let* $\{w^{k}\}$ *be the sequence generated by MHD*-*ALM*. *Then*, *for any* $w^{*}\in\mathcal{W}^{*}$ *and integer* $t\geq1$, *we have*

$$\bigl\Vert M\bigl(v^{t}-\hat{v}^{t}\bigr) \bigr\Vert _{H}^{2}\leq\frac{1}{c_{0}t} \bigl\Vert v^{0}-v^{*} \bigr\Vert _{H}^{2}, $$

*where* $c_{0}>0$ *is a constant*.

The term

$$\frac{1}{2\alpha t} \bigl\Vert v-v^{0} \bigr\Vert _{H}^{2} $$

on the right-hand side of (21) is used to measure the ergodic convergence rate of MHD-ALM. However, it is not only independent of the distance between the initial iterate $w^{0}$ and the solution set $\mathcal{W}^{*}$ but also hard to estimate due to the variable *v*. Therefore, inequality (21) is not a reasonable criterion to measure the nonergodic convergence rate of MHD-ALM. In the following, we shall give a refined result from the objective function and constraint condition of problem (1), which is more reasonable, accurate, and intuitive.

### Lemma 3.3

*Let* $\{w^{k}\}$ *be the sequence generated by MHD*-*ALM*. *Then*, *for any* $w\in\mathcal{W}$, *we have*

22 $$ \alpha\bigl(\theta(x)-\theta\bigl(\hat{x}^{k}\bigr)+ \bigl(w-\hat {w}^{k}\bigr)^{\top}F\bigl(\hat{w}^{k} \bigr)\bigr) \geq\frac{1}{2}\bigl( \bigl\Vert v-v^{k+1} \bigr\Vert ^{2}_{H}- \bigl\Vert v-v^{k} \bigr\Vert _{H}^{2}\bigr)+\frac{\alpha}{2} \bigl\Vert v^{k}-\hat{v}^{k} \bigr\Vert _{H}^{2}. $$

### Proof

The proof is similar to that of Lemma 3.1 in [28] and is omitted for brevity of this paper. This completes the proof. □

### Theorem 3.4

(Refined ergodic convergence rate)

*Let* $\{w^{k}\}$ *be the sequence generated by MHD*-*ALM*, $\{\hat{w}^{k}\}$ *be the sequence defined in* (15). *Set*

$$\bar{w}^{t}=\frac{1}{t}\sum_{k=0}^{t-1} \hat{w}^{k}. $$

*Then*, *for any integer* $t\geq1$, *there exists a constant* $c>0$ *such that*

$$\textstyle\begin{cases} |\theta(\bar{x}^{t})-\theta(x^{*})|\leq\frac{c}{2\alpha t} , \\ \|\mathcal{A}\bar{x}^{t}-b\|\leq\frac{c}{2\alpha t} . \end{cases} $$

### Proof

Choose $w^{*}=(x^{*},\lambda^{*})\in\mathcal{W}^{*}$. Then, for any $\lambda\in\mathcal{R}^{l}$, we have $\tilde{w}^{*}:=(x^{*},\lambda)\in \mathcal{W}$. From the definition of $F(w)$ in (9), we have

$$\begin{array}{c} {\left({\tilde{w}}^{*}-{\hat{w}}^{k}\right)}^{⊤}F\left({\hat{w}}^{k}\right)\\ \;={\left({\tilde{w}}^{*}-{\hat{w}}^{k}\right)}^{⊤}F\left({\tilde{w}}^{*}\right)\\ \;={\left(\begin{array}{c} x^{*}-{\hat{x}}^{k}\\ \lambda -{\hat{\lambda }}^{k} \end{array}\right)}^{⊤}\left(\begin{array}{c} -A^{⊤}\lambda \\ Ax^{*}-b \end{array}\right)\\ \;=-\lambda^{⊤}\left(Ax^{*}-A{\hat{x}}^{k}\right)\\ \;=\lambda^{⊤}\left(A{\hat{x}}^{k}-b\right), \end{array}$$

where the first equation follows from (10). Setting $w=\tilde{w}^{*}$ in (22), we get

$$\alpha\bigl(\theta\bigl(\hat{x}^{k}\bigr)-\theta\bigl(x^{*}\bigr)-\bigl( \tilde{w}^{*}-\hat{w}^{k}\bigr)^{\top}F\bigl( \hat{w}^{k}\bigr)\bigr) \leq\frac{1}{2}\bigl( \bigl\Vert \tilde{v}^{*}-v^{k} \bigr\Vert _{H}^{2}- \bigl\Vert \tilde{v}^{*}-v^{k+1} \bigr\Vert ^{2}_{H} \bigr)-\frac{\alpha}{2} \bigl\Vert v^{k}-\hat{v}^{k} \bigr\Vert _{H}^{2}. $$

Combining the above two inequalities gives

$$\alpha\bigl(\theta\bigl(\hat{x}^{k}\bigr)-\theta\bigl(x^{*}\bigr)- \lambda^{\top}\bigl(\mathcal{A}\hat{x}^{k}-b\bigr)\bigr) \leq \frac{1}{2}\bigl( \bigl\Vert \tilde{w}^{*}-w^{k} \bigr\Vert _{H}^{2}- \bigl\Vert \tilde{v}^{*}-v^{k+1} \bigr\Vert ^{2}_{H}\bigr)-\frac{\alpha}{2} \bigl\Vert v^{k}-\hat{v}^{k} \bigr\Vert _{H}^{2}. $$

Summing the above inequality from $k=0$ to $t-1$ yields

$$\sum_{k=0}^{t-1}\theta\bigl( \hat{x}^{k}\bigr)-t\theta\bigl(x^{*}\bigr)-\lambda^{\top}\Biggl( \mathcal {A}\sum_{k=0}^{t-1} \hat{x}^{k}-tb \Biggr)\leq\frac{1}{2\alpha} \bigl\Vert \tilde {v}^{*}-v^{0} \bigr\Vert _{H}^{2}. $$

Dividing both sides of the above inequality by *t*, we get

$$\frac{1}{t}\sum_{k=0}^{t-1}\theta \bigl(\hat{x}^{k}\bigr)-\theta\bigl(x^{*}\bigr)-\lambda^{\top}\bigl(\mathcal{A}\bar{x}^{t}-b\bigr)\leq\frac{1}{2\alpha t} \bigl\Vert \tilde{v}^{*}-v^{0} \bigr\Vert _{H}^{2}. $$

Then it follows from the convexity of $\theta_{i}$ ($i=1,2,3$) that

23 $$\theta \left({\bar{x}}^{t}\right)-\theta \left(x^{*}\right)-\lambda^{⊤}\left(A{\bar{x}}^{t}-b\right)\leq \frac{1}{2\alpha t}{∥\left(\begin{array}{c} y^{0}-y^{*}\\ \lambda^{0}-\lambda \end{array}\right)∥}_{H}^{2},$$

where $y^{0}=(x_{2}^{0},x_{3}^{0})$, $y^{*}=(x_{2}^{*},x_{3}^{*})$. Since (23) holds for any *λ*, we can set

$$\lambda=-\frac{\mathcal{A}\bar{x}^{t}-b}{\|\mathcal{A}\bar{x}^{t}-b\|}, $$

and consequently,

$$\theta \left({\bar{x}}^{t}\right)-\theta \left(x^{*}\right)+∥A{\bar{x}}^{t}-b∥\leq \frac{1}{2\alpha t}\underset{∥\lambda ∥\leq 1}{\sup}{∥\left(\begin{array}{c} y^{0}-y^{*}\\ \lambda^{0}-\lambda \end{array}\right)∥}_{H}^{2}.$$

Set

$$c=\underset{∥\lambda ∥\leq 1}{\sup}{∥\left(\begin{array}{c} y^{0}-y^{*}\\ \lambda^{0}-\lambda \end{array}\right)∥}_{H}^{2},$$

and we thus get

$$\theta\bigl(\bar{x}^{t}\bigr)-\theta\bigl(x^{*}\bigr)+ \bigl\Vert \mathcal{A}\bar{x}^{t}-b \bigr\Vert \leq\frac {c}{2\alpha t}. $$

Since $x^{*}\in\mathcal{X}^{*}$ (here $\mathcal{X}^{*}$ denotes the solution set of problem (1)), we have

$$\theta\bigl(\bar{x}^{t}\bigr)-\theta\bigl(x^{*}\bigr)\geq0. $$

Combining the above two inequalities gives

$$\textstyle\begin{cases} |\theta(\bar{x}^{t})-\theta(x^{*})|\leq\frac{c}{2\alpha t} , \\ \|\mathcal{A}\bar{x}^{t}-b\|\leq\frac{c}{2\alpha t} , \end{cases} $$

which completes the proof. □

As mentioned in Sect. 1, He *et al.* [12] used a simple example to show that the iterative scheme (6) may diverge for two-block separable convex programming. If we set $\theta _{1}=0$, $A_{1}=0$ in (1) and MHD-ALM, then MHD-ALM reduces to the method in [12]. In this case, the feasible set of *α* in [12] is (0,0.3670), the same as that of the method in [25] for three-block separable convex programming. Now we use this example given in [12] to show that: (i) larger values of $\alpha\in(0,2-\sqrt{2})$ can enhance the performance of MHD-ALM; (ii) MHD-ALM with $\alpha\geq2-\sqrt{2}\cong0.5858$ may diverge.

### Example 3.1

Consider the linear equation

24 $$ x_{2}+x_{3}=0. $$

Obviously, the linear equation (24) is a special case of problem (1) with the specifications: $\theta_{1}=\theta _{2}=\theta_{3}=0$, $A_{1}=0$, $A_{2}=A_{3}=1$, $b=0$, $\mathcal{X}_{1}=\mathcal {X}_{2}=\mathcal{X}_{3}=\mathcal{R}$. Due to $\theta_{1}=0$, $A_{1}=0$, in the following we do not consider the variable $x_{1}$. The solution set of the corresponding mixed variation inequalities is

$$\mathcal{W}^{*}=\bigl\{ \bigl(x_{2}^{*},x_{3}^{*},\lambda^{*} \bigr)|x_{2}^{*}+x_{3}^{*}=0,\lambda^{*}=0\bigr\} . $$

For MHD-ALM, we set $\beta=1$, the initial point $x_{2}^{0}=x_{3}^{0}=0$, $\lambda ^{0}=1$, and choose

$$\alpha\in\{0.20,0.21,0.22,\ldots,0.55\}. $$

The stopping criterion is set as

$$\max\bigl\{ \bigl\vert x_{2}^{k}+x_{3}^{k} \bigr\vert , \bigl\vert \lambda^{k} \bigr\vert \bigr\} \leq10^{-5}, $$

or the number of iterations exceeds 10,000.

**Figure 1:**
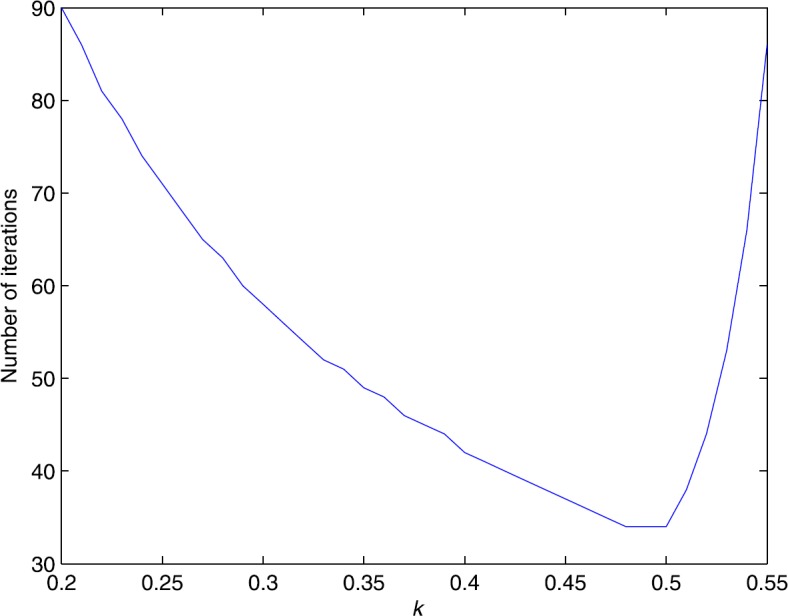
Sensitivity test on the step size *α*

The numerical results are graphically shown in Fig. 1, which illustrates that when $\alpha\leq0.5$, the number of iterations is descending with respect to *α*, while when $\alpha\in(0.5,0.55)$, the number of iterations increases quickly. Therefore, $\alpha=0.5$ is optimal for this problem, and larger values of *α* in its feasible set indeed can enhance the numerical performance of MHD-ALM. Of course, some extreme values, such as the values near the upper bound 0.5858, are not appropriate choices.

Now, we show that MHD-ALM may diverge for $\alpha\geq2-\sqrt{2}$. By some simple manipulations, the iterative scheme of (13) and (14) for problem (24) can be written in the following compact form:

25 $$\left(\begin{array}{c} x_{2}^{k+1}\\ x_{3}^{k+1}\\ \lambda^{k+1} \end{array}\right)=P(\alpha )\left(\begin{array}{c} x_{2}^{k}\\ x_{3}^{k}\\ \lambda^{k} \end{array}\right),$$

where

$$P(\alpha )=\left(\begin{array}{ccc} 1-\alpha & -\alpha & \alpha \\ -\alpha & 1-\alpha & \alpha \\ \alpha & \alpha & 1-2\alpha \end{array}\right).$$

Three eigenvalues of the matrix $P(\alpha)$ are

$$\lambda_{1}=1,\qquad \lambda_{2}=1-2\alpha+\sqrt{2}\alpha,\qquad \lambda_{3}=1-2\alpha -\sqrt{2}\alpha. $$

Now let us consider the following two cases:

(1) For any $\alpha>2-\sqrt{2}$, we have

$$\lambda_{3}=1-(2+\sqrt{2})\alpha< 1-(2+\sqrt{2}) (2-\sqrt{2})=-1. $$

Then $\rho(P(\alpha))>1$ for $\alpha>2-\sqrt{2}$, where $\rho(P(\alpha ))$ is the spectral radius of $P(\alpha)$. Hence, the iterative scheme (25) with $\alpha>2-\sqrt{2}$ is divergent for this problem.

(2) For $\alpha=2-\sqrt{2}$, by eigenvalue decomposition, the matrix $P(2-\sqrt{2})$ can be decomposed as

$$P(2-\sqrt{2})=VDV^{\top}, $$

where

$$V=\left(\begin{array}{ccc} -1/2 & -\sqrt{2}/2 & 1/2\\ -1/2 & \sqrt{2}/2 & 1/2\\ \sqrt{2}/2 & 0 & \sqrt{2}/2 \end{array}\right),\;D=\left(\begin{array}{ccc} -1 & 0 & 0\\ 0 & 1 & 0\\ 0 & 0 & 4\sqrt{2}-5 \end{array}\right).$$

Thus, by (25), we get

$$\left(\begin{array}{c} x_{2}^{k+1}\\ x_{3}^{k+1}\\ \lambda^{k+1} \end{array}\right)=\left(\begin{array}{c} -\frac{\sqrt{2}({\left(-1\right)}^{k}-{\left(4\sqrt{2}-5\right)}^{k})}{4}\\ -\frac{\sqrt{2}({\left(-1\right)}^{k}-{\left(4\sqrt{2}-5\right)}^{k})}{4}\\ \frac{{\left(-1\right)}^{k}}{2}+\frac{{\left(4\sqrt{2}-5\right)}^{k}}{2} \end{array}\right),$$

from which it holds that

$$\left(\begin{array}{c} x_{2}^{2k}\\ x_{3}^{2k}\\ \lambda^{2k} \end{array}\right)\to \left(\begin{array}{c} -\frac{\sqrt{2}}{4}\\ -\frac{\sqrt{2}}{4}\\ \frac{1}{2} \end{array}\right),\;\left(\begin{array}{c} x_{2}^{2k+1}\\ x_{3}^{2k+1}\\ \lambda^{2k+1} \end{array}\right)\to \left(\begin{array}{c} \frac{\sqrt{2}}{4}\\ \frac{\sqrt{2}}{4}\\ -\frac{1}{2} \end{array}\right).$$

Hence, the iterative scheme (25) with $\alpha=2-\sqrt{2}$ is also divergent for this problem. Overall, $2-\sqrt{2}$ is the optimal upper bound of the step size *α* in MHD-ALM.

Now let us consider some special cases and extensions of MHD-ALM:

(1) Problem (1) with $\theta_{1}=0$, $A_{1}=0$ reduces to two-block separable convex programming, which can be solved by MHD-ALM as follows:

26 $$ \textstyle\begin{cases} \tilde{x}_{2}^{k}= {\operatorname{argmin}}\{\mathcal{L}_{\beta}(x_{2},x_{3}^{k},\lambda ^{k})|x_{2}\in\mathcal{X}_{2}\}, \\ \tilde{x}_{3}^{k}= {\operatorname{argmin}}\{\mathcal{L}_{\beta}({x}_{2}^{k},x_{3},\lambda^{k})|x_{3}\in\mathcal{X}_{3}\}, \\ \tilde{\lambda}^{k}=\lambda^{k}-\beta(A_{2}\tilde{x}_{2}^{k}+A_{3}\tilde{x}_{3}^{k}-b), \end{cases} $$

and

27 $$ \textstyle\begin{cases} {x}_{2}^{k+1}=x_{2}^{k}-\alpha(x_{2}^{k}-\tilde{x}_{2}^{k}), \\ {x}_{3}^{k+1}=x_{3}^{k}-\alpha(x_{3}^{k}-\tilde{x}_{3}^{k}), \\ {\lambda}^{k+1}=\lambda^{k}-\alpha(\lambda^{k}-\tilde{\lambda}^{k}). \end{cases} $$

Since the iterative scheme (26), (27) is a special case of MHD-ALM, it is convergent for any $\alpha\in(0,2-\sqrt{2})$. Furthermore, by Example 3.1, $2-\sqrt{2}$ is the optimal upper bound of the constant step size *α* in (26), (27).

(2) Similarly, problem (1) with $\theta_{3}=0$, $A_{3}=0$ also reduces to two-block separable convex programming, which can be solved by MHD-ALM as follows:

28 $$ \textstyle\begin{cases} \tilde{x}_{1}^{k}= {\operatorname{argmin}}\{\mathcal{L}_{\beta}(x_{1},x_{2}^{k},\lambda ^{k})|x_{1}\in\mathcal{X}_{1}\}, \\ \tilde{x}_{2}^{k}= {\operatorname{argmin}}\{\mathcal{L}_{\beta}(\tilde {x}_{1}^{k},x_{2},\lambda^{k})|x_{2}\in\mathcal{X}_{2}\}, \\ \tilde{\lambda}^{k}=\lambda^{k}-\beta(A_{1}\tilde{x}_{1}^{k}+A_{2}\tilde{x}_{2}^{k}-b), \end{cases} $$

and

29 $$ \textstyle\begin{cases} {x}_{1}^{k+1}=\tilde{x}_{1}^{k}, \\ {x}_{2}^{k+1}=x_{2}^{k}-\alpha(x_{2}^{k}-\tilde{x}_{2}^{k}), \\ {\lambda}^{k+1}=\lambda^{k}-\alpha(\lambda^{k}-\tilde{\lambda}^{k}). \end{cases} $$

Following a similar analysis procedure, we can prove that the iterative scheme (28), (29) is convergent for any $\alpha\in(0,1)$.

(3) Extending MHD-ALM to solve four-block separable convex programming:

$$\min \Biggl\{ \sum_{i=1}^{4} \theta_{i}(x_{i})\Big|\sum_{i=1}^{4}A_{i}x_{i}=b,x_{i} \in\mathcal {X}_{i},i=1,2,3,4 \Biggr\} , $$

we can get the following iterative scheme:

30 $$ \textstyle\begin{cases} \tilde{x}_{1}^{k}= {\operatorname{argmin}}\{\mathcal{L}_{\beta}(x_{1},x_{2}^{k},x_{3}^{k},x_{4}^{k},\lambda^{k})|x_{1}\in\mathcal{X}_{1}\}, \\ \tilde{x}_{2}^{k}= {\operatorname{argmin}}\{\mathcal{L}_{\beta}(\tilde {x}_{1}^{k},x_{2},x_{3}^{k},x_{4}^{k},\lambda^{k})|x_{2}\in\mathcal{X}_{2}\}, \\ \tilde{x}_{3}^{k}= {\operatorname{argmin}}\{\mathcal{L}_{\beta}(\tilde {x}_{1}^{k},x_{2}^{k},x_{3},x_{4}^{k},\lambda^{k})|x_{3}\in\mathcal{X}_{3}\}, \\ \tilde{x}_{4}^{k}= {\operatorname{argmin}}\{\mathcal{L}_{\beta}(\tilde {x}_{1}^{k},x_{2}^{k},x_{3}^{k},x_{4},\lambda^{k})|x_{4}\in\mathcal{X}_{4}\}, \\ \tilde{\lambda}^{k}=\lambda^{k}-\beta(\mathcal{A}_{1}\tilde{x}^{k}-b), \end{cases} $$

and

31 $$ \textstyle\begin{cases} {x}_{1}^{k+1}=\tilde{x}_{1}^{k}, \\ {x}_{2}^{k+1}=x_{2}^{k}-\alpha(x_{2}^{k}-\tilde{x}_{2}^{k}), \\ {x}_{3}^{k+1}=x_{3}^{k}-\alpha(x_{3}^{k}-\tilde{x}_{3}^{k}), \\ {x}_{4}^{k+1}=x_{4}^{k}-\alpha(x_{4}^{k}-\tilde{x}_{4}^{k}), \\ {\lambda}^{k+1}=\lambda^{k}-\alpha(\lambda^{k}-\tilde{\lambda}^{k}). \end{cases} $$

With similar reasoning, we can prove that the iterative scheme (30), (31) is convergent for any $\alpha\in(0,2-\sqrt{3})$.

## Numerical results

In this section, we demonstrate the practical efficiency of MHD-ALM by applying it to recover low-rank and sparse components of matrices from incomplete and noisy observation. Furthermore, to give more insight into the behavior of MHD-ALM, we compare it with the full Jacobian decomposition of the augmented Lagrangian method (FJD-ALM) [12] and the proximal partially parallel splitting method with constant step size (PPPSM) [25]. All experiments are performed on a Pentium(R) Dual-Core CPU T4400@2.2 GHz PC with 4 GB of RAM running on 64-bit Windows operating system.

The mathematical model of recovering low-rank and sparse components of matrices from incomplete and noisy observation is [20]

32 $$ \begin{aligned} &\min_{L,S,U} \biggl\{ \Vert L \Vert _{*}+\tau \Vert S \Vert _{1}+ \frac{1}{2\mu} \bigl\Vert P_{\Omega}(U) \bigr\Vert ^{2} \biggr\} , \\ &\quad \mbox{s.t. } L+S+U=D, \end{aligned} $$

where $D\in\mathcal{R}^{p\times q}$ is a given matrix, $\tau>0$ is a balancing parameter, $\mu>0$ is a penalty parameter, $\Omega\subseteq\{ 1,2,\ldots,p\}\times\{1,2,\ldots,q\}$ is the index set of the observable entries of *D*, and $P_{\Omega}:\mathcal{R}^{p\times q}\rightarrow\mathcal{R}^{p\times q}$ is the projection operator defined by

$$\bigl[P_{\Omega}(X)\bigr]_{ij}= \textstyle\begin{cases} X_{ij},& \mbox{if } (i,j)\in\Omega, \\ 0,& \mbox{otherwise}, \end{cases}\displaystyle \quad 1\leq i\leq p, 1\leq j\leq q. $$

Problem (32) is a concrete model of the generic problem (1), and MHD-ALM is applicable. For this problem, the three minimization problems in (13) all admit closed-form solutions, which can be found in [20].

### Simulation example

We generate the synthetic data of (32) in the same way as [5, 20]. Specifically, let $L^{*}$, $S^{*}$ be the low-rank matrix, the sparse matrix, respectively, and rr, spr, and sr represent the ratios of the low-rank ratio of $L^{*}$ (i.e., $r/p$), the number of nonzero entries of $S^{*}$ (i.e., $\|S^{*}\|_{0}/(pq)$), and the observed entries (i.e., $|\Omega /(pq)$), respectively. The observed part of the matrix *D* is generated by the following Matlab scripts, in which *b* is the vectorization of *D*:

$$\begin{aligned}& \mathtt{X = randn(m,rr*m) * randn(rr*m,n); \qquad Omega = randperm(m*n); } \\& \mathtt{p = round(sr*m*n);\qquad Omega = Omega(1:p);\qquad Omega = Omega';} \\& \mathtt{Y = zeros(m,n);\qquad L = round\bigl(min(spr,sr)*m*n\bigr);\qquad nzp = Omega(1:L);} \\& \mathtt{Y(nzp) = \bigl(rand(L,1)*2-1\bigr)*500; b = X + Y; } \\& \mathtt{b = b(Omega);\qquad sigma = 0.001;\qquad b = b + sigma * randn(p,1); } \end{aligned}$$

In this experiment, we set $\tau=1/\sqrt{p}$, $\mu=\sqrt{p+\sqrt {8p\sigma}}/10$, $\beta=\frac{0.06|\Omega|}{\|P_{\Omega}(D)\|_{1}}$, the initial iterate $(L^{0},S^{0},U^{0},\lambda^{0})=(0,0,0,0)$, and use the stopping criterion

33 $$ \max \biggl\{ \frac{\|\tilde{L}^{k}-L^{k}\|}{1+\| L^{k}\|},\frac{\|\tilde{S}^{k}-S^{k}\|}{1+\|S^{k}\|} \biggr\} < 10^{-4}, $$

or the number of iterations exceeds 500.

The parameters in the three tested methods are listed as follows:

- FJD-ALM: $\alpha=0.38$.
- PPPSM: $S_{i}=0$, $H=\beta I$, $Q=I$, $\alpha=0.36$.
- MHD-ALM: $\alpha=0.5$.

In Tables 1 and 2, we report the numerical results of three tested methods, in which the number of iterations (denoted by ‘Iter.’), the elapsed CPU time in seconds (denoted by ‘Time’), the relative error of the recovered low-rank matrix, and the relative error of the recovered sparse matrix are reported when the stopping criterion (33) is satisfied.

**Table 1:** Numerical comparisons between different algorithms for $p=q=500$

rr	spr	sr	Method	Iter.	Time	$\frac{\|L^{k}-L^{*}\|}{\|L^{*}\|}$	$\frac{\|S^{k}-S^{*}\|}{\|S^{*}\|}$
0.05	0.05	0.9	FJD-ALM	97	7.42	5.7325e−04	9.7830e−05
			PPPSM	133	14.70	2.4034e−04	3.6358e−05
			MHD-ALM	44	5.17	7.5910e−04	1.5025e−04
0.05	0.05	0.6	FJD-ALM	120	11.03	1.6528e−03	1.3102e−04
			PPPSM	181	17.62	5.4329e−04	5.1513e−05
			MHD-ALM	75	6.76	1.7148e−03	1.1343e−04
0.1	0.1	0.9	FJD-ALM	127	11.39	2.2566e−03	2.2374e−04
			PPPSM	175	17.79	5.2215e−04	6.1755e−05
			MHD-ALM	80	6.86	1.5097e−03	1.5994e−04
0.1	0.1	0.8	FJD-ALM	188	17.45	4.2439e−03	2.7536e−04
			PPPSM	222	20.78	1.2697e−03	9.5339e−05
			MHD-ALM	119	10.15	2.8338e−03	1.9242e−04
0.1	0.15	0.9	FJD-ALM	209	20.18	3.9031e−03	2.3423e−04
			PPPSM	222	25.95	1.3444e−03	9.5133e−05
			MHD-ALM	120	12.37	3.2420e−03	2.0704e−04

**Table 2:** Numerical comparisons between different algorithms for $p=q=1000$

rr	spr	sr	Method	Iter.	Time	$\frac{\|L^{k}-L^{*}\|}{\|L^{*}\|}$	$\frac{\|S^{k}-S^{*}\|}{\|S^{*}\|}$
0.05	0.05	0.9	FJD-ALM	105	93.16	2.6808e−04	6.1154e−05
			PPPSM	129	131.08	9.9216e−05	2.3683e−05
			MHD-ALM	46	46.82	3.5053e−04	9.5144e−05
0.05	0.05	0.6	FJD-ALM	139	117.67	7.1439e−04	6.8845e−05
			PPPSM	147	138.08	2.1260e−04	3.4427e−05
			MHD-ALM	57	53.31	5.9883e−04	9.8047e−05
0.1	0.1	0.9	FJD-ALM	119	100.13	9.3082e−04	1.6381e−04
			PPPSM	188	179.91	2.3296e−04	4.2820e−05
			MHD-ALM	61	57.04	8.9591e−04	1.6198e−04
0.1	0.1	0.8	FJD-ALM	133	114.80	1.2760e−03	1.8083e−04
			PPPSM	192	182.46	3.7423e−04	5.2624e−05
			MHD-ALM	76	67.08	1.3497e−03	1.6979e−04
0.1	0.15	0.9	FJD-ALM	139	117.89	1.9819e−03	2.3040e−04
			PPPSM	206	188.47	4.4773e−04	5.7452e−05
			MHD-ALM	84	76.34	1.6396e−03	1.8795e−04

Numerical results in Tables 1 and 2 indicate that: (i) all methods successfully solved all the tested cases; (ii) both MHD-ALM and PPPSM perform better than FJD-ALM, and MHD-ALM performed the best. The reason maybe that FJD-ALM updates all the primal variables in a parallel manner, while PPPSM and MHD-ALM update $x_{2}$, $x_{3}$ based on the newest updated $x_{1}$ to accelerate the convergence speed. Furthermore, the step size *α* of MHD-ALM is larger than that of PPPSM, and the latter is larger than that of FJD-ALM. Therefore, larger values of *α* can enhance the efficiency of the corresponding method.

### Application example

In this subsection, we apply the proposed method to solve the video background extraction problem with missing and noisy data [29]. There is a video taken in an airport, which consists of 200 grayscale frames with each frame having $144\times176$ pixels. We need to separate its background and foreground. Vectorizing all frames of the video, we get a matrix $D\in\mathcal {R}^{25\text{,}344\times50}$, and each column represents a frame. Let $L, S\in \mathcal{R}^{25\text{,}344\times200}$ be the matrix representations of its background and foreground (i.e., the moving objects), respectively. Then the rank of *L* is equal to one exactly, and *S* should be sparse with only a small number of nonzero elements. We consider only a fraction entries of *D* can be observed, whose indices are collected in the index set Ω. Then the background extraction problem with missing and noisy data can be casted as problem (32). In the experiment, the parameters in MHD-ALM are set as $\alpha=0.5$, $\beta=\frac{0.005|\Omega|}{\|P_{\Omega}(D)\|_{1}}$, the parameters in (32) are set as $\tau= 1/\sqrt{p}$, $\mu=0.01$, and the initial iterate $(L^{0},S^{0},U^{0},\lambda^{0})=(0,0,0,0)$. We use the same stopping criterion as (33) with the tolerance 10^−2^.

**Figure 2:**
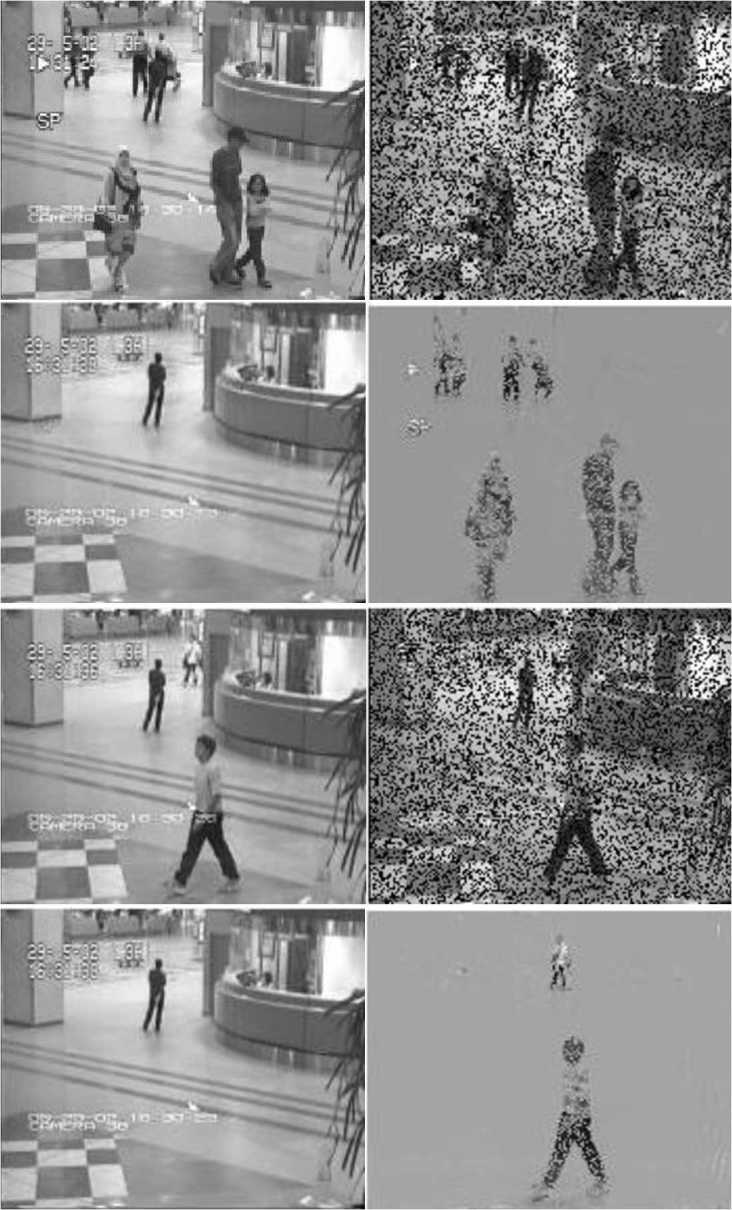
The 10th and 125th frames of the clean video and the corresponding corrupted frames with $\mathtt{sr}=0.7$ (the top and third lines); the extracted background and foreground by MHD-ALM (the second and fourth lines)

Figure 2 displays the separation results of the 10th and 125th frames of the video with $\mathtt{sr}=0.7$, which indicate that the proposed MHD-ALM successfully separates the background and foreground of the two frames.

## Conclusion

In this paper, a hybrid decomposition of the augmented Lagrangian method is proposed for three-block separable convex programming, whose most important characteristic is that its correction step adopts a constant step size. We showed that the optimal upper bound of the constant step size is $2-\sqrt{2}$. Preliminary numerical results indicate that the proposed method is more efficient than similar methods in the literature.

The following two issues deserve further researching: (i) Due to Condition 2.1 being only a sufficient condition to ensure the convergence of the ProAlo, is 1 the optimal upper bound of *α* in the iterative scheme (28), (29)? Similarly, is $2-\sqrt {2}$ the optimal upper bound of *α* in the iterative scheme (30), (31)? (ii) If we choose different step sizes for $x_{2}$, $x_{3}$, *λ* in the correction step of MHD-ALM, the feasible set of these step sizes needs more discussion.
